# Prevalence and Subtype Distribution of *Blastocystis* Infection in Patients with Diabetes Mellitus in Thailand

**DOI:** 10.3390/ijerph17238877

**Published:** 2020-11-29

**Authors:** Noppon Popruk, Satakamol Prasongwattana, Aongart Mahittikorn, Attakorn Palasuwan, Supaluk Popruk, Duangdao Palasuwan

**Affiliations:** 1 Department of Transfusion Medicine and Clinical Microbiology, Faculty of Allied Health Sciences, Chulalongkorn University, Bangkok 10330, Thailand; 6076752537@student.chula.ac.th; 2 Department of Nursing, Bang Pa-in Hospital, Bang Pa-in District, Phra Nakhon Si Ayutthaya 13160, Thailand; satakamon.j@hotmail.com; 3 Department of Protozoology, Faculty of Tropical Medicine, Mahidol University, Bangkok 10400, Thailand; aongart.mah@mahidol.ac.th; 4 Oxidation in Red Cell Disorders Research Unit, Department of Clinical Microscopy, Faculty of Allied Health Sciences, Chulalongkorn University, Bangkok 10330, Thailand; Attakorn.P@chula.ac.th

**Keywords:** *Blastocystis*, diabetes mellitus, SSU rRNA gene, subtypes

## Abstract

Diabetes mellitus (DM) is a major global public health problem with an increasing prevalence. DM increases the risk of infections caused by bacteria, fungi, viruses, and parasites. We examined the prevalence, subtypes, and risk factors of *Blastocystis* infection in patients with and without DM in central Thailand. Stool samples and questionnaires were obtained from 130 people in the DM group and 100 people in the non-DM group. *Blastocystis* infection was identified via a nested polymerase chain reaction and subtyped via sequencing of the partial small-subunit ribosomal RNA (SSU rRNA) gene. Analysis of potential risk factors was conducted via binary logistic regression. The overall prevalence of *Blastocystis* infection was 10.8%, including rates of 9% and 12.3% in the non-DM and DM groups, respectively. The most prevalent subtype was ST3, followed by ST1, and ST4. Factors that potentially increased the risk of *Blastocystis* infection include patients being >65 years old, the presence of DM, a DM duration of ≥10 years, a low level of education, and animal ownership. In conclusion, this is the first study of *Blastocystis* infection in DM, and a high prevalence was found among this population. Therefore, health education promoting sanitation and hygiene is necessary to reduce and prevent infection in the community.

## 1. Introduction

Intestinal parasitic infection has a global distribution, and its prevalence is especially high in developing countries. Simultaneously, these countries have increasingly higher levels of public health problems related to non-communicable diseases, such as cardiovascular diseases, cancers, chronic respiratory diseases, and diabetes mellitus (DM) [1]. People with diabetes may be more susceptible to infectious disease than those without diabetes. Both innate immune response defects (including neutrophil and macrophage dysfunction) and dysfunction of the adaptive immune response (including T cells) are believed to be responsible for immune system weakness against invading pathogens in people with diabetes [2]. DM is associated with increased rates of infection, especially those caused by bacteria [3,4,5]. A few studies of parasitic infection among people with diabetes have been reported to date [6]. These studies suggest that DM is significantly associated with the prevalence of intestinal parasites or common intestinal parasites, such as *Ascaris lumbricoides*, *Entamoeba histolytica*, *Giardia duodenalis*, and *Opisthorchis viverrini* [7,8,9]. Thailand has a rapidly increasing prevalence of type 2 DM (T2DM) [10,11]. Several studies on the prevalence of intestinal parasites in healthy subjects in Thailand have been reported [12,13,14]. Surprisingly, there is a paucity of information on *Blastocystis* sp., one of the most frequent protozoa found in humans.

*Blastocystis* sp. is an enteric protozoan found in both humans and animals with a worldwide distribution [15]. Because of its genetic heterogeneity, the genetic variants have been grouped into subtypes based on sequence similarity [16]. At present, 17 subtypes of *Blastocystis* sp. have been reported. Subtypes (ST)1–ST8 have been detected in both humans and animals. Conversely, ST9 has only been found in humans, while other subtypes have been found in animals. ST1–ST4 are the most prevalent *Blastocystis* subtypes found in humans [17]. The role of *Blastocystis* sp. as a human pathogen is unclear. Gastrointestinal symptoms such as diarrhea, abdominal pain, bloating and constipation, and extraintestinal disorders, such as cutaneous lesions, may be associated with *Blastocystis* infection [18,19,20]. In Thailand, studies on *Blastocystis* infection have been conducted in various groups in different community settings [21,22,23]. The prevalence of *Blastocystis* sp. has been reported to be as high as 45% in Thailand [24]. To date, no studies have been conducted on the prevalence of *Blastocystis* infection in patients with DM in the country.

Therefore, the present study investigated the prevalence and subtype distribution of *Blastocystis* infection in patients with diabetes using PCR-based methods. In addition, we also examined the associations between risk factors and *Blastocystis* infection.

## 2. Materials and Methods

### 2.1. Study Area

A cross-sectional study was conducted at primary health care hospitals between November 2019 and February 2020. This study focused on participants living in the Bang Pa-in district in the Phra Nakhon Si Ayutthaya province, which is located in central Thailand. The Bang Pa-in district is located approximately 64 km north of Bangkok. It is a semi-urban community with the second highest population after the Phra Nakhon Si Ayutthaya district. According to population-based health information system data, the local population in the fiscal year of 2019 was approximately 90,000, including 4000 people with diabetes. The most important river of the Phra Nakhon Si Ayutthaya province, namely Chao Phraya River, flows along this study area. Villagers living along the river use the water for agriculture, farming, and transportation. Furthermore, Bang Pa-in Industrial Estate requires raw water from the river for production. As a result, these activities may produce and discharge waste into water resources, including canals. These characteristics make this area suitable for conducting the study.

### 2.2. Study Population and Study Design

To increase the efficiency of population recruitment in this study, we used a primary health care database. The primary health care system is the smallest and most effective infrastructure of the Thai health care system [25]. The study population consisted of 130 participants with DM and 100 participants without DM. The criteria for participant selection were as follows: older than 40 years of age, native resident of the district, and no use of anthelmintic/antiprotozoal drugs for at least 3 months prior to enrolment. All participants were asked to provide fresh stool samples for parasite detection. All participants gave written informed consent to participate in this study, and they were directly interviewed to obtain basic demographic information using questionnaires. The study protocol was reviewed and approved by the Research Ethics Review Committee for Research Involving Human Research Participants, Health Sciences Group, Chulalongkorn University (certificate of approval number: 231/2562).

### 2.3. Stool Collection and Processing

After providing informed consent, the participants were asked to complete a brief questionnaire. Then, the study participants were given a stool collection kit and standard instructions on proper and safe collection. The participants were asked to provide one stool sample. All stool samples were shipped under cool conditions to the laboratory of Protozoology Department, Faculty of Tropical Medicine, Mahidol University (Thailand) within 4–6 h after evacuation for processing. To detect *Blastocystis* sp., the stool samples were aliquoted and frozen in −20 °C until further DNA extraction.

### 2.4. DNA Extraction and Nested PCR Amplification

All stool samples were extracted using a QIAamp Fast DNA Stool Mini Kit (Qiagen, Hilden, Germany) following the manufacturer’s protocol. The extracted DNA was stored at −20 °C until use. To identify *Blastocystis* sp., the 1.1-kb SSU rRNA gene was detected using nested PCR. RD3 (5′-GGGATCCTGATCCTTCCGCAGGTTCACCTAC-3′) and RD5 (5′-GGAAGCTTATCTGGTTGATCCTGCCAGTA-3′) were the external primers used for primary PCR [26], and an internal set of forward (5′-GGAGGTAGTGAC AATAAATC-3′) and reverse primers (5′-ACTAGGAATTCCTCGTTCATG-3′) was used for secondary PCR [27]. Each 25-µL reaction mixture contained 1× PCR buffer, 1.5 mM MgCl_2_, 0.2 mM dNTPs, 1 µM each primer and 2.5 U of *Taq* DNA polymerase (Thermo Fisher Scientific, Waltham, MA, USA). PCR products were separated by electrophoresis in 1.5% agarose gel in the presence of ethidium bromide, visualized using ultraviolet transillumination, and photographed.

### 2.5. Sequencing and Phylogenetic Analysis

The positive PCR products of the 1100-bp fragment of the *Blastocystis* SSU rRNA gene were sequenced in two directions using appropriate internal primers on an ABI 3730xl automated DNA sequencer by Bio Basic Inc. (Bukit Batok, Singapore). *Blastocystis* subtypes were identified using a BLAST search of the National Center for Biotechnology Information database (https://blast.ncbi.nlm.nih.gov/Blast.cgi). The raw nucleotide sequences and 18 reference sequences were edited manually using BioEdit v.7.2.5 Software (Ibis Biosciences, Carlsbad, CA, USA), and a multiple alignment was performed using ClustalW (Table 1). Finally, MEGA version 6 software was used for phylogenetic analysis. The best model to account for the evolution of the DNA sequences was the Hasegawa–Kishino–Yano model with gamma distribution. A phylogenetic tree was constructed with the maximum likelihood method and tested with 1000 bootstrap replicates. The 25 nucleotide sequences generated in this study were deposited in GenBank under the following accession numbers: MT330258–MT330260, MT330263, MT330265–MT330267, MT330269–MT330277, and MT947108–MT947116.

### 2.6. Statistical Analysis

Descriptive analysis was used to describe the characteristics, prevalence, and subtype distribution. The chi-squared test was used to analyze the potential risk factors for *Blastocystis* infection. Odds ratios (ORs) and the corresponding 95% confidence intervals (CIs) were used to measure the degree of association between *Blastocystis* infection and potential risk factors. All statistical analyses were performed using IBM SPSS Statistics for Windows, version 22 (IBM Corp., Armonk, NY, USA), and *p* < 0.05 was considered statistically significant.

## 3. Results

### 3.1. Basic Characteristics of the Individuals

The mean age of the study participants was 66.7 years (range, 44–88), and 56.5% (130/230) of the participants had DM. Most study participants were female (66.5%). The participant characteristics of the DM and non-DM groups are presented in Table 2. There was a slightly significant difference in the level of education between the non-DM and DM groups.

### 3.2. The Prevalence and Subtype of Blastocystis sp.

The overall prevalence of *Blastocystis* infection was 10.8% (25/230). The prevalence rates of *Blastocystis* infection were 9% (9/100) and 12.3% (16/130) in the non-DM and DM groups, respectively (Table 3). There was no association between participant characteristics and *Blastocystis* infection. Male sex (OR = 1.373, 95% CI = 0.586–3.218), age ≥ 65 years (OR = 1.630, 95% CI = 0.673–3.949), presence of DM (OR = 1.419, 95% CI = 0.599–3.36), DM duration ≥ 10 years (OR = 1.439, 95% CI = 0.499–4.153), less than secondary school education (OR = 1.525, 95% CI = 0.338–6.877), and presence of animals in the household (OR = 1.337, 95% CI = 0.565–3.167) tended to increase the risk of *Blastocystis* infection, but none of the associations were significant. 

The 25 nucleotide sequences of the partial SSU rRNA gene in the present study displayed an extremely high similarity (≥98%) to existing sequences of *Blastocystis* reported in GenBank (Table 4), and they were identified as three distinct subtypes: ST1, ST3, and ST4. *Blastocystis* ST3 was the most prevalent subtype found in both groups, followed by ST1 and ST4. *Blastocystis* ST4 was only found in one sample in the non-DM group (Table 5). 

### 3.3. Phylogenetic Analysis

**Figure 1:**
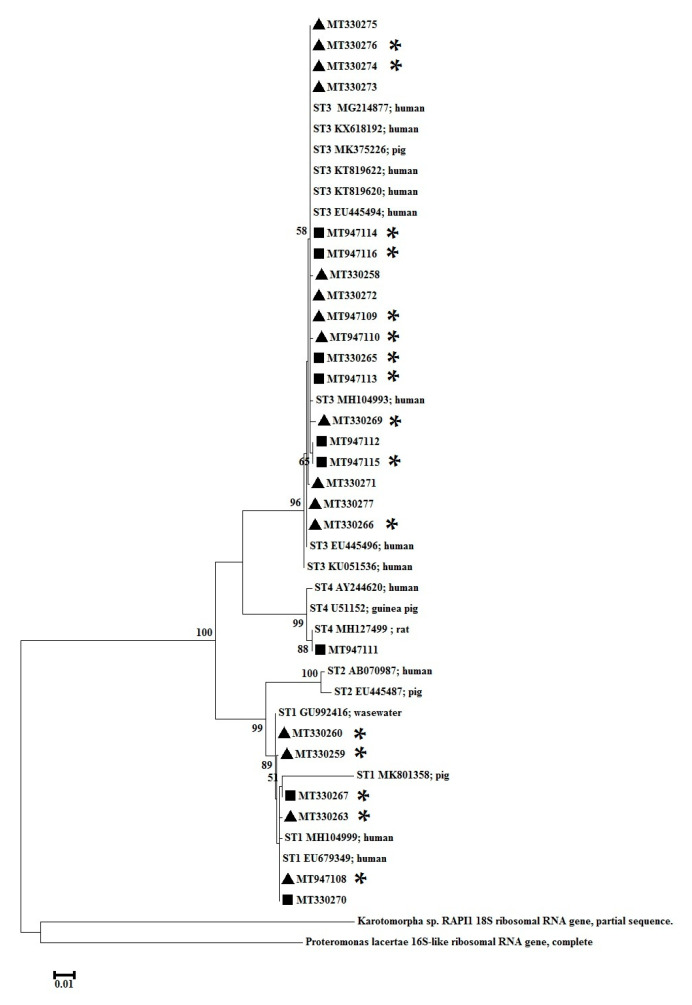
Maximum likelihood analysis of the *Blastocystis* SSU rRNA (small subunit ribosomal RNA) gene based on the general time reversible model. The outgroup sequences were *Proteromonas lacertae* (U37108) and *Karotomorpha* sp. (DQ431242). Symbol ▲—Diabetes DM, ■—Non-DM and *—Presence of animals in the household.

We performed a phylogenetic analysis of 25 nucleotide sequences of *Blastocystis*-positive samples compared with the 1–4 reference subtype sequences in GenBank. The sequence of the *Blastocystis* ST4-positive sample from the non-DM group was closely related to rat-derived sequences in GenBank, as presented in Figure 1.

## 4. Discussion

*Blastocystis* sp. has been reported in humans worldwide. In the present study, our data, obtained using nested PCR, revealed a high prevalence of *Blastocystis* infection among subjects with and without DM. However, the prevalence of *Blastocystis* infection in this study was lower than that in prior studies conducted in asymptomatic individuals in Thailand [22,41,42] but was similar to findings in other developing countries [43,44]. The highest prevalence of *Blastocystis* infection of 45.2% was reported in children in an orphanage in Pathum Thani province, Thailand [25]. Conversely, the prevalence was low in developed countries such as Japan (0.5–1%) [45] and Singapore (3.3%) [27]. Prevalence varies between countries and between regions within the same country. This variation could be related to the health status of the study population, geographic distribution, and detection method.

Our finding that ST3 was the predominant *Blastocystis* subtype was similar to the results of previously reported studies in Thailand [24,42,46,47] and other countries such as Japan, Bangladesh, Pakistan [38], and China [48]. Contrarily, other studies found that *Blastocystis* ST1 was the most predominant subtype [49,50]. *Blastocystis* ST3 is most commonly found in humans in Europe, Africa, Asia, and Australia, whereas the most common subtype in America is ST1 [51]. Nevertheless, *Blastocystis* ST1–ST4 have been identified as the most common subtypes in humans [18,21]. In this study, *Blastocystis* ST1 was the second-most common subtype, and it was found in almost all individuals with animals in their homes. It has been suggested that *Blastocystis* ST1 is associated with zoonotic transmission to humans [52]. Our finding indicates that *Blastocystis* ST1 detected in these people might have been caused by exposure to animal stools. *Blastocystis* ST4 was found in only one sample in the non-DM group. Our result is consistent with a previous study reporting a low prevalence of ST4 in Africa, America, and Asia [51].

In our study, 230 completed questionnaires were used to assess possible risk factors for *Blastocystis* infection. *Blastocystis* infection was not significantly related to any participant characteristics. This result is consistent with previous findings in patients with DM undergoing hemodialysis [53]. Meanwhile, another study found that *Blastocystis* infection was significantly more prevalent in patients with DM than in those without DM [54]. Interestingly, our results illustrated that most infected individuals consume safe drinking water. On the contrary, several studies indicated that *Blastocystis* infection was significantly associated with the quality of drinking water [55,56]. This discrepancy could be attributed to other potential factors associated with *Blastocystis* infection, such as sanitation and hygiene practices. However, the fecal–oral route is considered to be the major mode of transmission of this protozoan [19]. The prevalence of *Blastocystis* infection among the two groups was not statistically different in the present study. The findings of other studies indicate that type 2 diabetes in humans is associated with compositional changes in the intestinal microbiota that decrease the abundance of some universal butyrate-producing bacteria and increase the likelihood of various opportunistic pathogens [57,58]. *Blastocystis* can release proteases that affect the integrity of the epithelial tissue and possibly facilitate colonization by other intestinal pathogens and changes in the intestinal microbiota diversity and composition [59,60]. 

The phylogenetic tree indicated that the 25 nucleotide sequences in the same subtype clusters had good bootstrap support and belonged to three subtypes: ST1, ST3, and ST4. One nucleotide sequence with ST4 in this study was located on the same branch as the reference sequence of wild rats in Japan [39]. Unlike *Blastocystis* ST1 and ST3, which are commonly found in humans, rodents have been suggested to be the reservoir host of *Blastocystis* ST4 [61]. Our findings suggest that *Blastocystis* ST4 may have been transmitted from rodents to this participant.

## 5. Conclusions

This report is the first regarding the prevalence and subtype distribution of *Blastocystis* sp. in patients with DM in Thailand. This protozoan was more prevalent in the DM than in the non-DM group. Although, we observed no association between *Blastocystis* infection and potential risk factors (participant characteristics), the potential risk factors for *Blastocystis* infection, including DM and close contact with animals, should not be excluded. To better understand the association between potential risk factors and *Blastocystis* infection, it will be necessary to increase the sample size, examine a wide variety of populations, including immunocompromised people, and expand the survey area. 

## 6. Limitations

First, we recruited study participants based on the presence of diabetes diagnosed by the Thai health care system. The DM and non-DM groups differed at baseline concerning the glycemic control profile. The difference in glycemic control between the groups may have affected the risk of infection in these individuals. Although the control group consisted of people without diabetes, the presence of other underlying diseases may have affected the study results. Second, we did not obtain other data about the participants such as medical history or confidential information because such data cannot be obtained without patient permission or legal authorization. Moreover, the sample size was a limitation in this study as well. 

## Figures and Tables

**Table 1:** GenBank references for the *Blastocystis* subtypes (ST)1–ST4 sequences used to construct a phylogenetic tree.

Subtype	Accession Number	Host
1	EU679349	Human [28]
	GU992416	Wastewater [29]
	MH104999	Human [30]
	MK801358	Pig [31]
2	AB070987	Human [32]
	EU445487	Pig [33]
3	EU445494	Human [33]
	EU445496	Human [33]
	KT819620	Human [34]
	KT819622	Human [34]
	KU051536	Human [35]
	KX618192	Human [36]
	MG214877	Human (unpublished)
	MH104993	Human [30]
	MK375226	Pig [37]
4	AY244620	Human [38]
	MH127499	Rat [39]
	U51152	Guinea pig [40]

**Table 2:** Characteristics of study participants with diabetes mellitus (DM, *n* = 130) and without diabetes mellitus (Non-DM, *n* = 100).

Characteristics		DM *n* = 130 (%)	Non-DM *n* = 100 (%)	Total *n* = 230 (%)	*p*
Gender	Male	43/130 (33.1%)	34/100 (34%)	77/230 (33.5%)	0.883
	Female	87/130 (66.9%)	66/100 (66%)	153/230 (66.5%)	
Age	<65 years	54/130 (41.5%)	43/100 (43%)	97/230 (42.2%)	0.824
	≥65 years	76/130 (58.5%)	57/100 (57%)	133/230 (57.8%)	
Level of education	Low (≤primary school)	120/130 (92.3%)	84/100 (84%)	204/230 (88.7%)	0.049 *
	High (≥secondary school)	10/130 (7.7%)	16/100 (16%)	26/230 (11.3%)	
Employed	No	66/130 (50.8%)	58/100 (58%)	124/230 (53.9%)	0.275
	Yes	64/130 (49.2%)	42/100 (42%)	106/230 (46.1%)	
Source of drinking water	Treated water (bottled and tap water)	118/130 (90.8%)	96/100 (96%)	214/230 (93%)	0.122
	Untreated water (surface and rainwater)	12/130 (9.2%)	4/100 (4%)	16/230 (7%)	
Animals in the household	No	53/130 (40.8%)	44/100 (44%)	97/230 (42.2%)	0.623
	Yes	77/130 (59.2%)	56/100 (56%)	133/230 (57.8%)	

* *p* < 0.05; DM, diabetes mellitus.

**Table 3:** Association between participant characteristics and *Blastocystis* infection in present study.

Characteristics		Number of Examined	% Infected	OR (95% CI) *
Gender	Male	77	13 (10/77)	1.373 (0.586–3.218)
	Female	153	9.8 (15/153)	1
Age	<65 years	97	8.2 (8/97)	1
	≥65 years	133	12.8 (17/133)	1.63 (0.673–3.949)
DM status	Non-DM	100	9 (9/100)	1
	DM	130	12.3 (16/130)	1.419 (0.599–3.36)
Duration of DM	≤10 years	83	10.8 (9/83)	1
	>10 years	47	14.9 (7/47)	1.439 (0.499–4.153)
Education status	Low (≤primary school)	204	11.3 (23/204)	1.525 (0.338–6.877)
	High (≥secondary school)	26	7.7 (2/26)	1
Employed	No	124	12.1 (15/124)	1.321 (0.567–3.078)
	Yes	106	9.4 (10/106)	1
Source of drinking water	Treated water (bottled and tap water)	214	11.2 (24/214)	1.895 (0.239–14.99)
	Untreated water (surface and rainwater)	16	6.3 (1/16)	1
Presence of animals in household	Yes	133	12 (16/133)	1.337 (0.565–3.167)
	No	97	9.3 (9/97)	1

* Binary logistic regression, *p* > 0.05. DM, diabetes mellitus; OR, odds ratio; CI, confidence interval.

**Table 4:** Accession numbers of positive samples used in the phylogenetic reconstruction in this study.

GenBank Accession No.	Subtype	Group (DM/Non-DM)	Query Cover	Sequence Similarity (%)	Similar GenBank Reference Sequence
MT330258	3	DM	97%	99.53	KT819620
MT330259	1	DM	98%	99.44	MH104999
MT330260	1	DM	97%	99.44	GU992416
MT330263	1	DM	98%	99.07	EU679349
MT330265	3	Non-DM	99%	98.44	KT819620
MT330266	3	DM	99%	98.52	KT819620
MT330267	1	Non-DM	98%	98.98	MK801358
MT330269	3	DM	98%	99.17	KT819620
MT330270	1	Non-DM	99%	98.99	MK801358
MT330271	3	DM	98%	99.44	KT819620
MT330272	3	DM	98%	99.26	KT819620
MT330273	3	DM	97%	99.81	KX618192
MT330274	3	DM	97%	99.81	KT819620
MT330275	3	DM	97%	99.81	KT819620
MT330276	3	DM	97%	99.63	KX618192
MT330277	3	DM	97%	99.54	MN914073
MT947108	1	DM	100%	99.5	MH104999
MT947109	3	DM	100%	99.9	MH104993
MT947110	3	DM	100%	99.26	KT819622
MT947111	4	Non-DM	100%	99.8	MH127499
MT947112	3	Non-DM	100%	99.8	MG214877
MT947113	3	Non-DM	100%	99.9	MK375226
MT947114	3	Non-DM	100%	100	MK375226
MT947115	3	Non-DM	100%	99.8	MK375226
MT947116	3	Non-DM	100%	99.9	MK375226

DM, diabetes mellitus.

**Table 5:** Subtype distribution in the diabetes mellitus (DM) and non-DM groups.

Group	Subtype Distribution Positive Case/Total No. (%)			Total
	ST1	ST3	ST4	
DM	4/130 (3%)	12/130 (9.3%)	0/130 (0%)	16/130 (12.3%)
Non-DM	2/100 (2%)	6/100 (6%)	1/100 (1%)	9/100 (9%)

DM, diabetes mellitus.
